# Zebrafish regulatory genomic resources for disease modelling and regeneration

**DOI:** 10.1242/dmm.050280

**Published:** 2023-08-02

**Authors:** Ada Jimenez Gonzalez, Damir Baranasic, Ferenc Müller

**Affiliations:** ^1^ Institute of Cancer and Genomic Sciences, Birmingham Centre for Genome Biology, College of Medical and Dental Sciences, University of Birmingham, Birmingham B15 2TT, UK; ^2^ Institute of Clinical Sciences, Faculty of Medicine, Imperial College London, Hammersmith Hospital Campus, London SW7 2AZ, UK; ^3^ MRC London Institute of Medical Sciences, London W12 0NN, UK; ^4^ Division of Electronics, Ruđer Bošković Institute, Bijenička cesta 54, 10000 Zagreb, Croatia

**Keywords:** Disease modelling, Genomic resources, Regeneration

## Abstract

In the past decades, the zebrafish has become a disease model with increasing popularity owing to its advantages that include fast development, easy genetic manipulation, simplicity for imaging, and sharing conserved disease-associated genes and pathways with those of human. In parallel, studies of disease mechanisms are increasingly focusing on non-coding mutations, which require genome annotation maps of regulatory elements, such as enhancers and promoters. In line with this, genomic resources for zebrafish research are expanding, producing a variety of genomic data that help in defining regulatory elements and their conservation between zebrafish and humans. Here, we discuss recent developments in generating functional annotation maps for regulatory elements of the zebrafish genome and how this can be applied to human diseases. We highlight community-driven developments, such as DANIO-CODE, in generating a centralised and standardised catalogue of zebrafish genomics data and functional annotations; consider the advantages and limitations of current annotation maps; and offer considerations for interpreting and integrating existing maps with comparative genomics tools. We also discuss the need for developing standardised genomics protocols and bioinformatic pipelines and provide suggestions for the development of analysis and visualisation tools that will integrate various multiomic bulk sequencing data together with fast-expanding data on single-cell methods, such as single-cell assay for transposase-accessible chromatin with sequencing. Such integration tools are essential to exploit the multiomic chromatin characterisation offered by bulk genomics together with the cell-type resolution offered by emerging single-cell methods. Together, these advances will build an expansive toolkit for interrogating the mechanisms of human disease in zebrafish.

## Teaser

**Summary:** Regulatory genomics resources for zebrafish need to be expanded to fulfil this model's potential in exploring the genetics of human disease. Bulk and single-cell genomics resources offer complementary advantages, which are best exploited by improving data integration.

## Zebrafish models in studying gene regulation underlying disease and regeneration

Zebrafish is a vertebrate model used by nearly 1000 laboratories worldwide (Mullins et al., 2021). The increased popularity of zebrafish is due to the ease of genome manipulation, and developmental and scalable phenotyping of transparent embryos (Howe et al., 2017). Furthermore, most human genes associated with disease have at least one zebrafish orthologue (Howe et al., 2013), making zebrafish an attractive genetic experimental model to test the function of disease genes and their regulation (Kettleborough et al., 2013). The high conservation of coding genes is accompanied by conserved key pathways and their downstream targets (Amatruda and Zon, 1999; van der Vaart et al., 2012) and supports the utility of zebrafish in genetic analyses.

Genome-wide association studies show that a large proportion of disease-associated gene variants occur in non-coding regions that include *cis*-regulatory elements (CREs; see Glossary, Box 1), such as promoters (Box 1) and enhancers (Box 1) (Watanabe et al., 2019). Disease-associated non-coding variants, their mechanisms of gene expression regulation, and their role in disease are still mostly unknown, requiring extensive functional annotation (Box 1) and characterisation (Alsheikh et al., 2022). Mutations in enhancers can affect gene expression through altered transcription factor (TF) binding. For example, NEUROD1, a TF associated with diabetes, was found to bind less efficiently to a variant enhancer sequence in pancreatic islets, leading to reduced enhancer activity and, as a result, increased susceptibility to type 2 diabetes in East Asian populations (Pasquali et al., 2014). Mutations can also impact enhancer tethering to its target gene promoter by enhancer looping (Box 1), as shown in Alzheimer's disease (Kikuchi et al., 2019). In this case, single-nucleotide polymorphisms (SNPs) disrupt the binding sites of CCCTC-binding factor (CTCF; Box 2), a key factor involved in the formation of chromatin loops, leading to misexpression of the disease-associated genes, *GATS* (*CASTOR3P*) and *PILRB*. Variants can also add regulatory functionality through the creation of new promoters, as was the case for the variant found upstream of the globin genes in α-thalassemia patients (De Gobbi et al., 2006). Besides point mutations, larger-scale chromosomal rearrangements can disrupt promoter targeting by misplacing enhancers and deregulating the expression of non-target genes, as seen in neuroblastomas  (Helmsauer et al., 2020).

**Box 1. Glossary**

***Cis*-regulatory elements (CREs):** non-coding DNA sequences, such as promoters and enhancers, that regulate the transcription of neighbouring genes for proper spatiotemporal expression.

**Constitutive orphan predicted element (COPE):** regulatory element open throughout development but without having an active chromatin mark at any stage.

**Dynamic orphan predicted element (DOPE):** regulatory element active at some point in development but without having an active chromatin mark at any stage.

**ENCODE:** encyclopaedia of elements aiming to characterise the regulatory elements within the human genome.

**Enhancer:** *cis*-regulatory element that activates gene expression in a spatiotemporal manner by interacting with transcription factors and other proteins that promote the assembly of the transcription machinery at the promoter of a gene. These sequences can be located either upstream, downstream or within the introns of a gene, often exhibiting long-range effects. Enhancers can be inactive, active or primed. This can be determined based on the presence of particular histone marks (e.g. H3K4me1 and/or H3K27ac) and the levels of chromatin accessibility (Box 2).

**Enhancer looping:** folding of chromatin to bring distally located enhancers to the vicinity of a promoter in order to activate gene expression.

**Enhancer trap:** reporter assay consisting of a construct containing a minimal promoter and a reporter gene that is randomly integrated within the genome. The aim of this approach is to detect the spatial activity of a nearby enhancer activating the expression of the reporter gene through interaction with the inserted promoter.

**Functional annotation:** assignment of functional and biological information to a DNA sequence.

**ModENCODE:** model organism encyclopaedia of DNA elements characterising the regulatory elements in the *Caenorhabditis elegans* and *Drosophila* genomes.

**nf-core:** community-created resource aiming for the development of pipelines following standardised guidelines under the Nextflow system. This allows reproducibility between analyses, using the same tools and their versions.

**Nuclease-deficient Cas9 variant (dCas9):** Cas9 variant with a mutated catalytic domain that remains inactive. This ‘dead’ Cas9 conserves its target-specific binding activity, and it is used in CRISPR interference assays, allowing gene knockdown, or in gene activation approaches when linked to transcriptional activators.

**Predicted ATAC-seq-supported developmental regulatory elements (PADREs):** regulatory elements defined with ATAC-seq chromatin accessibility data and their function determined from ChIP-seq data integration of key histone modifications and predicted by the computational tool ChromHMM.

**Promoter:** *cis*-regulatory element instructing where transcription will initiate. It contains binding sites for general transcription factors, which are part of the transcription pre-initiation complex together with e RNA polymerase II.

**Safe harbour landing sites:** regions of the genome in which the integration of an ectopic DNA sequence does not disrupt the activity of the host genome.

**Self-organising map (SOM):** artificial neural network used to cluster multidimensional data.

**Syntenic anchors:** conserved non-repetitive sequences in two different genomes showing a high degree of similarity.

**Synteny:** blocks of syntenic anchors appearing in the same order in a given chromosome between organisms.

**Box 2. Chromatin features and technologies to identify them**

**Chromatin features and technical approaches**

**Transcription start site (TSS):** position within the genomic sequence in which the RNA polymerase starts transcribing a gene. TSS positions should be used for the identification of promoters. TSSs can be defined through cap analysis of gene expression (CAGE-seq). CAGE-seq is a genomic approach that allows accurate recognition of gene TSSs and analysis of the transcriptome. This approach relies on specifically sequencing the 5′ end of capped only RNA, thus allowing determination of the exact position from which these RNAs are transcribed.

**Enhancer RNAs (eRNAs):** type of long non-coding RNA transcribed from an active enhancer region that can be detected through CAGE-seq signal. The majority of eRNAs are transcribed in opposite directions from both DNA strands (i.e. bidirectionally transcribed).

**Chromatin accessibility:** this term refers to the extent to which chromatin is less condensed and therefore available or ‘open’ for transcription factors and other regulatory proteins to interact with it. The degree of accessibility is associated with the gene regulatory activity of a particular sequence, with open regions being found in active enhancers or promoters. As open chromatin is easily accessible by enzymes like transposases, the openness of a particular region of the chromatin can be determined through assays such as assay for transposase-accessible chromatin with sequencing (ATAC-seq). ATAC-seq uses a hyperactive mutant transposase to incorporate sequencing adaptors while simultaneously cleaving the DNA.

**Histone modifications:** post-translational modifications of histone tails, also known as histone marks, that are often associated with changes in chromatin regulatory activity. These modifications are traditionally detected by chromatin immunoprecipitation with sequencing (ChIP-seq) using specific antibodies for these marks to pull down their bound DNA. CUT&RUN (cleavage under targets and release using nuclease) is an alternative method that recognises protein–DNA interactions using a protein A–Tn5 transposase fusion approach that specifically targets the antibody-bound chromatin. Unlike ChIP-seq, this method allows for extremely low-input materials, avoids crosslinking and the crosslinking-related artifacts, and requires a lower number of sequencing reads, reducing costs. FitCUT&RUN is a variation of the CUT&RUN approach using Fc fragment of immunoglobulin G tagging. This is an antibody-free approach that addresses the problem of a lack of ChIP-seq- and CUT&RUN-compatible antibodies. Below, we summarise the properties of a few of these modifications that have been proved to be related to *cis*-regulatory elements.

- H3K4me3: histone mark predominantly found at gene promoters often associated with the expression of the marked gene.
- H3K4me1: histone mark associated with active or primed enhancers leading to gene expression of their target genes.
- H3K27ac: histone mark found in active enhancers and promoters associated with active expression.
- H3K27me3: histone mark linked to a particular example of transcriptional silencing known as polycomb repression and associated with chromatin compaction. This mark helps maintain the correct expression patterns of genes during development and cell differentiation.

**Chromatin interaction topology and technical approaches**

**Topology-defining boundaries:** genomic regions that define the three-dimensional organisation of the genome by acting as insulators or barriers.

**Topologically associating domain (TAD):** genomic region in which sequences show a high degree of interaction compared to the loci outside the structure. TADs are demarcated by topology-defining boundaries. These can be determined through chromosome conformation capture approaches.

**CCCTC-binding factor (CTCF):** zinc-finger protein that binds to a highly conserved sequence and acts as a transcriptional activator, repressor or promoter–enhancer insulator. CTCF is often found at TAD boundaries and it is thought to play a key role in forming and maintaining these structures. CTCF binding can be detected with ChIP-seq.

**Cohesin:** protein complex forming a ring-shaped structure that promotes proper chromosome segregation during mitosis and mediates DNA looping, therefore contributing to promoter–enhancer interactions and gene expression regulation.

**Chromosome conformation capture:** molecular technique that allows analysis of the spatial organisation of the chromatin within the nucleus.

- **4C-seq:** derivative of the chromosome conformation capture approach. 4C-seq enables studying all the genomic regions interacting with a selected locus.
- **Hi-C:** chromosome conformation capture-based technology that enables the genome-wide study of the three-dimensional architecture of the chromatin, including TADs, by measuring the frequency of interactions between DNA fragments.

Zebrafish embryos – with their ease of transgenesis and evolutionary conserved *cis*-regulatory logic during development – allow *in vivo* reporter assays to detect the effects of human-disease-associated, non-coding elements (Ghiasvand et al., 2011; Rada-Iglesias et al., 2011; Smemo et al., 2012; Pasquali et al., 2014; Kramer et al., 2022; Ferre-Fernández et al., 2022). These assays rely on extreme sequence conservation of candidate regulatory elements, which are enriched in developmental regulator genes often associated with disease (Elgar, 2009; Harmston et al., 2013; Pérez-Rico et al., 2017; Polychronopoulos et al., 2017). For example, the deletion of a highly conserved zebrafish enhancer of *gata2a* mimics dysregulated haematopoiesis characteristic of GATA2 deficiency in humans (Dobrzycki et al., 2020). This disease model was used in combination with single-cell RNA sequencing (RNA-seq) and single-cell assay for transposase-accessible chromatin with sequencing (ATAC-seq) to reveal that the lack of *gata2a* favours the erythroid lineage by reducing the accessibility of certain TFs in myeloid cells (Mahony et al., 2023). Furthermore, a dual fluorescence reporter system has recently been developed, which can simultaneously report the effects of SNPs alongside a common variant in conserved enhancers to verify their potential transcriptional impact (Bhatia et al., 2021). Even in cases in which sequence conservation is not sufficient to detect homologous enhancers between fish and human, conserved TF binding sites may predict shared function (Wong et al., 2020). Transgenic manipulation of enhancers with conserved TF binding site combinations in zebrafish has pinpointed a shared mechanism for pancreatic hypoplasia caused by mutations in the human pancreatic regulatory elements (Bordeira-Carriço et al., 2022).

Unlike humans, who can readily regenerate their skin, liver and fingertips, but not whole limbs or full organs, zebrafish can completely regenerate their spinal cord, brain, heart, kidney and fins. The regenerative mechanisms of these organs differ, yet they often include the reactivation of developmental pathways (reviewed in Goldman and Poss, 2020). With the emergence of regeneration cell atlases generated by single-cell transcriptomics (Wang et al., 2020; Hu et al., 2022; Jimenez et al., 2022), regeneration genetics is expected to increasingly utilise zebrafish in seeking vertebrate-conserved mechanisms (Suzuki et al., 2019), with the promise of potentially inducing gene reactivation in human organs for regenerative therapies (Goldman and Poss, 2020). Such mechanisms likely include regeneration-responsive enhancers in the heart, fin, retina (Kang et al., 2016; Hoang et al., 2020; Thompson et al., 2020) and inner ear hair cells (Jimenez et al., 2022).

## Gene manipulation strategies benefit from annotated *cis*-regulatory elements and chromatin maps

Annotation of enhancers and promoters is key to the development of genetic manipulation technology. Spatio-temporally controlled transgenic gain-of-function models require annotated, functionally validated CREs (Ertzer et al., 2007; Yuan et al., 2018; Liu et al., 2020). Loss-of-function models can also benefit from spatio-temporally controlled manipulation of endogenous genes, such as tissue-specific knockout using the Cre/lox system (Mukherjee and Liao, 2018; Hans et al., 2021) or cell-type-specific transgenic expression of genome-editing tools, such as Cas9 and associated guide RNAs (Ablain et al., 2015; Yin et al., 2015). Controlled activation of these transgenic effectors avoids early embryonic lethality, reduces pleiotropy, and offers precise modelling of disease states that result from tissue- and time-specific regulation of genes.

Annotation of promoters helps design reagents for transcriptional inhibition, such as the guide RNAs for either promoter deletion or for targeting nuclease-deficient Cas9 variant (dCas9; Box 1) to promoters, which offers an alternative to genetic lesions and to morpholino knockdown (Long et al., 2015). Such strategies critically depend on precise annotation of transcription start sites (TSSs; Box 2) that need to be targeted for efficient inhibition of gene expression by dCas9 (Baranasic et al., 2022).

Annotation of the genome regulatory landscape has additional benefits when considering loci for transgenesis. The chromatin environment in which a transgene lands can influence how efficiently it is expressed (called position effect). This is particularly important for transposon-mediated transgenesis, which predominantly occurs in active genomic regions (Vrljicak et al., 2016). To avoid position effects, safe harbour landing sites (Box 1) should be identified, which then can be targeted by site-specific recombination (Mosimann et al., 2013; Roberts et al., 2014). To detect such sites, maps of accessible chromatin and chromatin interaction topology (Box 2) can be informative. For the latter, zebrafish maps of chromatin interactions generated by Hi-C (Box 2) (Kaaij et al., 2018; Yang et al., 2020; Wike et al., 2021), and maps of interaction boundaries informed by CTCF- (Franke et al., 2021) and Cohesin- (Meier et al., 2018) binding sites (Box 2), offer guidance.

Zebrafish disease models benefit from *in vivo* labelling of cells and lineages, which enables *in vivo* tracking and sorting of specific cell populations. Although enhancer traps (Box 1) can identify a range of cell lineage markers (Kikuta et al., 2007; Kawakami et al., 2010), they depend on the serendipitous targeting of candidate loci. Cas9 knock-in of fluorescent reporters (reviewed in Liu et al., 2019) is a promising alternative (Kimura et al., 2014) to transposon transgenesis for cell labelling. This also benefits from annotated CREs for the precise control of fluorescent reporters or of Gal4 drivers (Kawakami et al., 2016).

## State-of-the-art zebrafish regulatory genomics resources and the challenges ahead

**Fig. 1.**
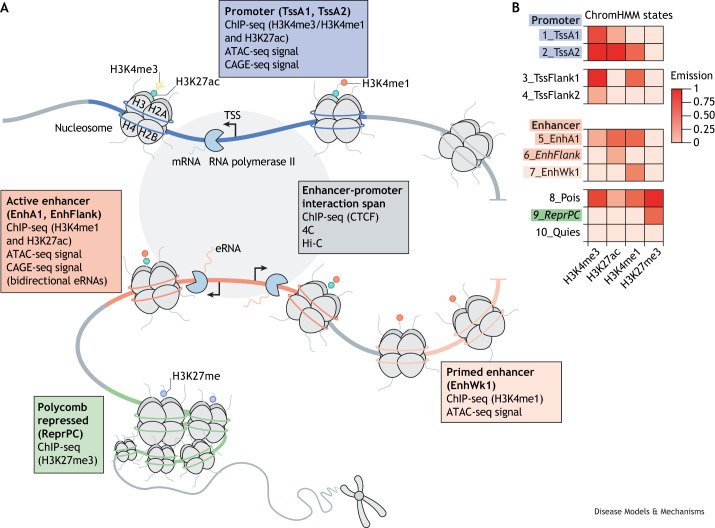
**The main features of regulatory elements as determined by DANIO-CODE.** (A) Regulatory elements defined by PADREs in DANIO-CODE are identified by ATAC-seq, which assesses chromatin accessibility, and ChIP-seq, which assesses histone modifications, and their function (i.e. level of active transcription) is predicted by the computational tool ChromHMM. This approach has allowed the identification and characterisation of candidate regulatory regions within the zebrafish genome, as shown in the schematic. Further considerations from the DANIO-CODE-available data are also included here. Within the promoter region, transcription of mRNA by RNA polymerase II occurs at the accessible TSS that is defined using CAGE-seq. The example promoters shown, TssA1 and TssA2, are accessible due to histone methylation (H3Kme3 or H3Kme1) and acetylation (H3K27ac), which make the chromatin less condensed. From the active enhancer region, eRNA is bidirectionally transcribed, which is also detected by CAGE-seq. The active enhancers shown, EnhA1 and EnhFlank, have H3Kme1 and H3K27ac histone modifications. Prior to activation, enhancers can exist in a primed state, known as primed enhancers, such as EnhWk1, which is associated with the H3Kme1 histone mark only. Promoter–enhancer interaction spans are detected by chromosome conformation capture techniques, 4C-seq and Hi-C, that can analyse interactions between genomic regions and the three-dimensional architecture of the chromatin. ChIP-seq can also be used to detect CTCFs, which are zinc-finger proteins that bind to highly conserved sequences and act as promoter–enhancer insulators. Finally, transcription can be repressed in polycomb-repressed regions by specific histone marks, such as H3K27me3 for ReprPC, which make the chromatin more condensed. (B) ChromHMM states have been included on the right for further reference (Baranasic et al., 2022). Each of the states are characterized by different levels of histone marks. The left side of this panel shows the different biologically relevant functions assigned to these states. ATAC-seq, assay for transposase-accessible chromatin with sequencing; CAGE-seq, cap analysis of gene expression; ChIP-seq, chromatin immunoprecipitation with sequencing; CTCF, CCCTC-binding factor; EnhA1, active enhancer 1; EnhFlank, enhancer flanking; EnhWk1, weak enhancer; eRNA, enhancer RNA; PADRE, predicted ATAC-seq-supported developmental regulatory element; Pois, poised; Quies, quiescent; ReprPC, repressed polycomb; TSS, transcription start site; TssA1/2, active transcription start site 1/2; TssFlank1/2, TSS flanking.

**Fig. 2.**
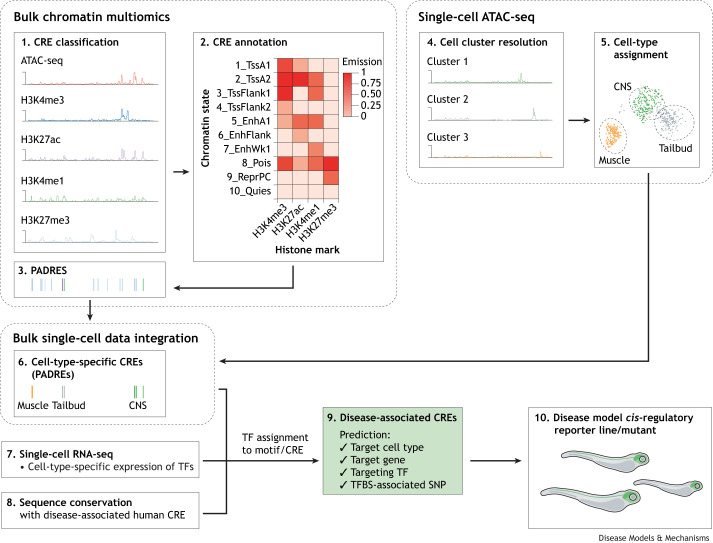
**Proposed integration pipeline for bulk chromatin and single-cell multiomics.** The numbers in fields indicate the flow of proposed data integration pipelines for zebrafish regulatory genomics. Bulk chromatin multiomics integrates CRE classification information from ATAC-seq data (1) with CRE annotation information from ChIP-seq data to predict regulatory element function using the computational tool ChromHMM (2) (Baranasic et al., 2022). This feeds predicted developmental regulatory elements into PADREs (3). Single-cell ATAC-seq can then provide further cell cluster resolution (4) to allow cell-type assignment (5) (McGarvey et al., 2022). Integration of the bulk and single-cell data will reveal cell-type-specific CREs within PADREs (6). Alongside this, single-cell RNA-seq can reveal cell-type-specific TF binding (7), and sequence conservation tracks can identify disease-associated human CREs (8). Once the cell type, target gene, TF and TFBS have been defined (9), a mutant reporter zebrafish line can be generated to function as a model of a human disease (10). CNS, central nervous system; CRE, *cis*-regulatory element; EnhA1, active enhancer 1; EnhFlank, enhancer flanking; EnhWk1, weak enhancer; PADRE, predicted ATAC-seq-supported developmental regulatory element; Pois, poised; Quies, quiescent; ReprPC, repressed polycomb; sc-ATAC-seq, single-cell ATAC-seq; sc-RNA-seq, single-cell RNA sequencing; SNP, single-nucleotide polymorphism; TF, transcription factor; TFBS, transcription factor binding site; TssA1/2, active transcription start site 1/2; TssFlank1/2, TSS flanking.

Expansion of zebrafish modelling of gene regulatory mechanisms in disease and regeneration relies on exploring and understanding the degree of sequence similarity between zebrafish and human regulatory elements. To assess the degree of conservation between fish and mammals, the regulatory element repertoire of zebrafish needs extensive mapping, similar to that achieved by large multinational mapping programmes such as ENCODE (Box 1) in humans (ENCODE Project Consortium, 2004). ENCODE and modENCODE (Box 1) (Brown and Celniker, 2015) significantly improved annotation of regulatory elements in human and key animal models. Recognising the need for generation of similar regulatory genomics resources, the zebrafish user community established the international network DANIO-CODE. DANIO-CODE collated and reanalysed ∼1800 genomics datasets by standardised pipelines, making them publicly available in the DANIO-CODE Data Coordination Center (DCC; https://danio-code.zfin.org). The DCC, representing 38 developmental stages, 21 assay types and 34 tissues, allows the sharing of raw and analysed data with public visualisation tracks. DANIO-CODE has functionally annotated 140,000 developmental candidate regulatory non-coding elements to comprehensively identify candidate promoters and enhancers by combining histone modification marks with ATAC-seq-supported open chromatin (Box 2) during zebrafish development. These predicted ATAC-seq-supported developmental regulatory elements (PADREs; Box 1) include annotations of precisely defined developmental promoteromes, including an alternative promoter catalogue verified by cap analysis of gene expression (CAGE-seq; Box 2). The PADREs have been classified into functionally distinct subcategories (Fig. 1), and their cell-type specificity has been predicted by integration with enhancer annotations emerging from developmental single-cell ATAC-seq (Fig. 2) (McGarvey et al., 2022). Additionally, detection of enhancer RNAs (eRNAs; Box 2) (Andersson et al., 2014) by analysing CAGE-seq data and sequence conservation tracks [e.g. cyprinid Phastcons (Chen et al., 2019)] may support the identification of enhancer candidates. Guidance on DANIO-CODE resources is provided in Box 3. DANIO-CODE resources will be beneficial for exploring the regulatory genome architecture of disease-associated genes and for the manipulation of their transcription. However, limitations and challenges remain that can be overcome with advances in technology and data integration.

**Box 3. Guidance on DANIO-CODE resources**

The DANIO-CODE Data Coordination Center (https://danio-code.zfin.org/) enables data visualisation and access to datasets with publicly available track hubs in the UCSC Genome Browser with two versions of the zebrafish genome assembly (danRer10 and danRer11). The DANIO-CODE atlas is based on strict thresholds to call regulatory regions to minimise background. Therefore, to expand enhancer discovery, users ought to explore the underlying signals (Fig. 1) and manually curate potentially missing regulatory elements. The list below includes a brief description of the information provided by the DANIO-CODE Track Hub collection, but please refer to DANIO-CODE Track Hub for complete documentation:

- Tracks for individual assay types:
  - ATAC-seq
  - Bisulfite sequencing (BS-seq)
  - CAGE-seq
  - ChIP-seq
  - Hi-C
  - Micrococcal nuclease digestion with deep sequencing (MNase-seq)
  - RNA-seq
- Cell types: tracks for the cell-type assignment to PADREs using single-cell ATAC-seq data.
- Consensus promoters: the DANIO-CODE Promoterome Atlas provides identified consensus promoters.
- Conservation and CRISPR targets: the conservation tracks and CRISPR targets tracks were kindly provided by Shawn Burgess at the National Human Genome Research Institute. These tracks mimic the public ZebrafishGenomics track hub.
- COPEs (Box 1) and pooled DOPEs (Box 1): this collection contains two tracks with regions that have an ATAC-seq signal, but without observable CRE-associated chromatin marks.
- Distal PADRE SOM (Box 1) classes: this collection contains tracks with PADREs that are not near promoter regions, clustered based on their patterns of openness throughout development.
- Enhancer validation: a collection of tracks to validate enhancer elements.
- H3K27ac ensembles: clusters of early developmental PADREs with uninterrupted H3K27ac signal connecting them.
- Mouse H3K27me3 on zebrafish coordinates: a signal of virtual whole-embryo mouse H3K27me3 ChIP-seq data on mapped zebrafish coordinates (mm_H3K27me3) and the annotations of the respective projected coordinates in mouse (mouse coordinates).
- Stage types: annotation tracks for regulatory elements defined by different methods.

Below we list the available DANIO-CODE resources, with detailed video tutorials on how to use them and contribute to them:

- Videos with tutorials and example usages of the resource: https://youtube.com/playlist?list=PLiWQCe7dGqm6AtA0oP7qIaEQNa-7Z7fh5
  - DANIO-CODE Track Hub for UCSC Genome Browser for danRer10: http://genome.ucsc.edu/cgi-bin/hgTracks?db=danRer10&hubUrl=https://danio-code.zfin.org/trackhub/DANIO-CODE.hub.txt
  - DANIO-CODE Track Hub for UCSC Genome Browser for danRer11: http://genome.ucsc.edu/cgi-bin/hgTracks?db=danRer11&hubUrl=https://danio-code.zfin.org/trackhub/DANIO-CODE.hub.txt
- Session for WashU Epigenome Browser: https://github.com/DANIO-CODE/DANIO-CODE_Data_analysis/tree/master/Figures/Figure1#figure-1c=
- Motif Activity Response Analysis (MARA): https://ismara.unibas.ch
  - DANIO-CODE results: https://ismara.unibas.ch/danio-code
- Regulatory motifs and regulatory site annotations: https://swissregulon.unibas.ch/sr/downloads
- Code repository for DANIO-CODE processing pipelines: https://gitlab.com/danio-code
- Code repository for data analysis in this paper: https://github.com/DANIO-CODE/DANIO-CODE_Data_analysis

### Expanding and refining the non-coding function elements of the genome

In DANIO-CODE, the candidate regulatory elements are classified by a limited number of chromatin marks available (Fig. 1 and Box 2). Functional annotations and epigenome mapping need to be refined with more marks, including those reflecting sites of active transcription [i.e. H3K36me3 (Zhang et al., 2018)] or those mapping heterochromatin [H3K9me2/3 (Laue et al., 2019)]. Additionally, there are limitations in detecting dynamic TF binding to regulatory elements in small cell number lineages owing to difficulties in using crosslinked chromatin for chromatin immunoprecipitation with sequencing (ChIP-seq; Box 2). Technologies such as CUT&RUN (Box 2) and FitCUT&RUN (Box 2) address these limitations, and there are increasing examples of successfully implementing them in zebrafish (Akdogan-Ozdilek et al., 2022; Wang et al., 2022; Truong et al., 2023). Besides the single-cell-based approaches, bulk RNA-seq data can also be interrogated for investigating TF activity dynamics that may inform targeting motifs in conserved regulatory elements. This can be done by the ISMARA tool (Table 1) (Balwierz et al., 2014), which has recently been applied to zebrafish promoter data (Baranasic et al., 2022). ISMARA has also been used to identify type 2 diabetes-associated regulatory elements in humans and to validate these results in zebrafish (Kirchner et al., 2016; Mattis et al., 2023). Similar human-disease-relevant studies can be achieved with the implementation of these emerging tools and technologies.

**Table 1.**
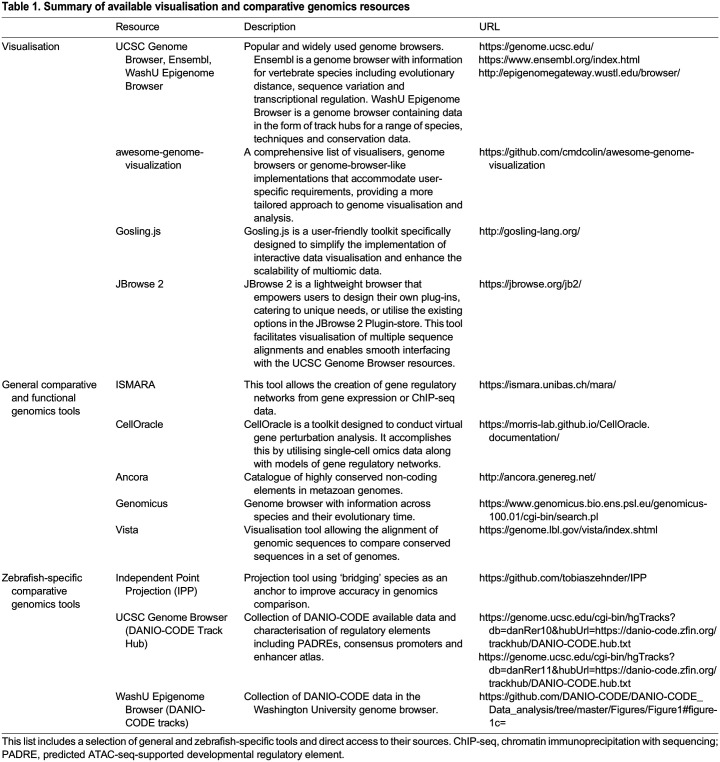
Summary of available visualisation and comparative genomics resources

### Enhancer–target gene matchmaking

A key challenge in the interpretation of function of disease-associated non-coding variants is the difficulty in identifying the correct target genes of the enhancer. Enhancers can act up to two megabases away from their targets (Long et al., 2016) and can reside in introns of unaffected bystander genes (Lettice et al., 2003). Promoter–enhancer targeting predictions have been made (Clément et al., 2020), and promoter–enhancer interaction maps generated from 4C-seq (Box 2) data may help in distinguishing targets at sufficiently high resolution. However, 4C-seq data are only available for a selected set of long-range regulated genes in DANIO-CODE. Zebrafish Hi-C data identified zebrafish topologically associating domains (TADs; Box 2), which are minable in the UCSC Genome Browser (Table 1) or in HiGlass (https://www.4dnucleome.org/). Together with TAD-boundary-associated CTCF-binding site maps (Box 2), this approach may inform promoter–enhancer interaction span.

### Species conservation tracks inform human disease biology

To enhance the applicability of the DANIO-CODE project to human disease, we must be able to identify homologous or functional equivalents to enhancers associated with human disease. This can be achieved by the integration of PADREs with annotated vertebrate conservation tracks to capture highly syntenic conserved sequences. Manual curation of conservation profiles can be improved with tools that allow non-syntenic comparisons. Several comparative genomics tools are listed in Table 1. The VISTA toolkit (Frazer et al., 2004) was used to predict disease-associated regulatory variants that were then functionally tested *in vivo*, and identified novel enhancers associated with the craniofacial abnormality Pierre Robin sequence (Bhatia et al., 2015). The Ancora database holds a catalogue of highly conserved non-coding elements in vertebrate genomes and visualises their density distribution. The results in density profiles may help in identifying their genomic targeting range (Engström et al., 2008). The conservation information from Ancora identifies potential transcriptional targets of a signalling pathway. This principle was demonstrated when it identified an enhancer that was targeted by bone morphogenetic protein (BMP) signaling, associated with neurological disorders (Zhang et al., 2020). The Genomicus browser (Nguyen et al., 2022) offers homology prediction and synteny (Box 1) analysis for studying the genetic basis of diseases in zebrafish. For example, synteny analysis informed the development of a zebrafish model of poikiloderma with neutropenia that closely recapitulated the human syndrome (Colombo et al., 2015). Synteny information also provided new insights into Krabbe's disease pathogenesis upon molecular cloning and knockdown of galactocerebrosidase in zebrafish (Zizioli et al., 2014), and for investigating glycogen storage in a zebrafish model of Pompe disease (Bragato et al., 2020). To improve detection of non-alignable enhancers, a multispecies comparison approach was developed called Independent Point Projection (IPP; Baranasic et al., 2022), which improves the resolution of synteny by increasing the number of syntenic anchors (Box 1) between fish and mammals (Table 1). This tool narrows the search space for enhancers that may not be alignable but share TF binding site composition, thus increasing the predictability of functional equivalence between candidate CREs in different species. Although this approach is not yet available as a web tool, it is available in GitHub for application and further development (Table 1).

### Expanding the zebrafish reference genome

Zebrafish regulatory resources need to be continually improved to serve as an up-to-date resource for the user community. Adding enhancers identified in zebrafish adult tissues (Yang et al., 2020) to the DANIO-CODE database is in progress at the time of writing this article. Furthermore, integration of recently acquired high-resolution chromatin conformation datasets and fast-expanding non-coding RNA annotations is also planned by the DANIO-CODE partners. The zebrafish reference genome is in its 11th iteration (Genome Reference Consortium Zebrafish Build 11 GRCz11); however, an end-to-end full-length reference genome has yet to be achieved. To this end, long-read sequencing offers high-confidence assembly of repeat-rich sequences, as was shown by the finalised human genome (Nurk et al., 2022). In zebrafish, long-read sequencing was applied to improve the poorly assembled chromosome 4 and has filled some gaps in the current reference genome (Yang et al., 2020; Chernyavskaya et al., 2022; Hadzhiev et al., 2023).

The zebrafish regulatory genomics resources will also benefit from improved standardisation of pipelines. The nf-core (Box 1) pipeline development project (Ewels et al., 2020) is being adapted by AQUA-FAANG (https://www.aqua-faang.eu/), which aims to annotate regulatory elements in farmed teleosts, including the cyprinid common carp. When published, AQUA-FAANG will offer lucrative teleost comparative genomic resources for zebrafish laboratories.

## Expanding and integrating single-cell and multiomic data

Bulk data have predominantly contributed to zebrafish regulatory genomics resources, which are far from comprehensive and need to be expanded. The bulk-data-based atlases will inevitably miss regulatory elements that are active in highly dynamic, stage-specific cells or in lineages with small cell numbers (Fig. 2). Combining the increasing number of single-cell open chromatin atlases and cell-type- and physiological-context-specific atlases, such as those for regeneration (Jimenez et al., 2022; McGarvey et al., 2022; Sur et al., 2023 preprint; Lange et al., 2023 preprint), with lower-resolution but more granular multiomic bulk data will improve cell-type resolution of regulatory annotations. However, until more single-cell data are accumulated and integrated, users are encouraged to critically browse the DANIO-CODE collection for biochemical chromatin features that were not interrogated by single-cell tools.

Thus, there remains the need to dissect the contribution of individual cells and lineages to emerging functional annotations from bulk tissue and from heterogeneous sorted cells. Integration of bulk and sorted cell data with single-cell ATAC data allows deconvolution, which can estimate the cell-type proportion and its contribution to the bulk data. Packages are available for bulk RNA-seq deconvolution (see Avila Cobos et al., 2020), but deconvoluting single-cell ATAC-seq still needs computational tool development. Popular genome browsers, such as the UCSC Genome Browser, Ensembl genome browser and WashU Epigenetic Browser (Table 1), are optimised for bulk data (Cunningham et al., 2022; Li et al., 2022; Nassar et al., 2023). For a compendium of further genomics analysis and visualisation tools see Table 1, which also includes a user-friendly toolkit, Gosling.js (http://gosling-lang.org/) (Lyi et al., 2022), and the lightweight browser JBrowse 2 (Diesh et al., 2023) offering expansion by user plug-ins.

A particularly pressing limitation of zebrafish regulatory annotations is the general lack of high-quality antibodies recognising zebrafish TFs and other chromatin-associated proteins. However, single-cell RNA-seq data offer high-resolution analysis of TF activities, which, when integrated with single-cell chromatin accessibility (Box 2) data, can link TFs to their enhancer targets, as has recently been demonstrated using CellOracle (Table 1) in zebrafish and with a related approach in mouse embryos (Fig. 2) (Argelaguet et al., 2022 preprint; Kamimoto et al., 2023).

Taken together, multiomic data integration tools, such as CellOracle, and yet-to-be-developed data visualisation tools will be necessary to maximise output from bulk and single-cell genomics and to generate more comprehensive regulatory genomic atlases for disease modelling and other applications (Fig. 2). Such new tools will be needed to combine the advantages of single-cell ATAC in cell-type resolution and sensitivity of feature detection with the advantages of bulk regulatory genomics. Until major breakthroughs are made in single-cell technologies for chromatin interrogation, it is expected that atlases with accurate prediction of regulatory elements' function, target gene, TF targeting, and cell-type and stage specificity will emerge by combining bulk and single-cell chromatin and transcriptome annotations (Fig. 2).

## Concluding remarks

Zebrafish regulatory genomics has not yet advanced disease modelling to the same degree as other aspects of zebrafish genetics. However, it is expected that improving genomic regulatory resources will lead to better understanding of the molecular pathways and their transcriptional targets involved in disease. In summary, profiling functional non-coding elements offers support in several ways. First, it enables more refined control of gene editing and transgenesis tools developed for disease and regeneration models. Second, the information derived from regulatory elements and their activities allows the identification, characterisation and manipulation of specific disease-associated cell populations. Last, dissecting regulatory element function and sequence and chromatin regulatory determinants can contribute to molecular understanding of conserved human disease and regeneration mechanisms and may, in the future, support treatment and diagnosis development.
